# Supplementary material for: Acceptance and compliance with micronutrient powder (MNP) among children aged 6–23 months in northern Nigeria
Source: PLOS Glob Public Health. 2022 Oct 17;2(10):e0000961. doi: 10.1371/journal.pgph.0000961 (PMC10022258; doi:10.1371/journal.pgph.0000961)
Supplement: S1 Dataset — (ZIP) [file pgph.0000961.s008.zip › S8_Dataset/Direct Observations.zip!/Direct Observations/220615_K_J_SU_1_DO.pdf]

1.1.1 Instrument F –Direct Observation Form

<<Instrument F Direct Observation Form>>
**PI Name:** PragyaMathema,Stephen Kodish<br>**Study Title:** Formative research to develop an infant and young child feeding (IYCF) intervention with micronutrient powder (MNP) for children aged 6 – 23 months<br>**PI Version/Date:** Version1.0/January 24, 2015

Date:22/06/2015 Location:Hillinde

Data Collector: Samira Usman

Main goals of this direct observation are to answer the following questions:
1. In this household, what would be the best way(s) to give nutritional supplements to young children? Why?<br><br>2. In this household, describe the food sharing that you observe throughout the meal. Are there any predictable patterns?<br><br>3. In this household, who is favored and how is this favoritism expressed during meal preparation and eating?

Household Composition Table (Fill out as you do data collection)

INITIALS	SEX (M/F)	AGE (estimated)	Relationship to household	Description
MM	F	35yrs	Mother	Orange wrapper blue head tie, tall and dark complexion
BB	M	17months	Baby/son	Red shirt
SW	F	25yrs	Brothers wife	Blue and brown wrapper
OW	F	57yrs	Second mother in-law	Green and milk lace wrapper and head tie
MI	F	59yrs	Mother in-law	Ankara wrapper top and green head tie
SB	M	4months	Cousin to the baby am observing	Naked baby
DI	F	16yrs	Daughter in-law	Average height yellow and black Ankara wrapper, top head tie
FF	F	45yrs	Father	Tall, dark wearing milk shadda

HDI	M	20yrs	Husband of the daughter in-law (first son)	Green top and trousers (shadda) average height
TB	M	15yrs	Son	Young boy wearing black trousers and blue shirt
SB	F	8yrs	son	Brown shirt and black short
SG	F	6yrs	Daughter	Red skirt and blue short with brown cap
SGS	F	4yrs	Daughter	Green and black skirt with black pants

TIME	ACTOR INITIALS	RECIPIENT INITIALS	WHAT HAPPENED	FOOD	Reactivity
8:35	MM	BB	Mother breast feeding the baby		
8:40	BB		Baby playing		
8:45	OW,MM,SH		Removing groundnut back cover and putting the seed in a calabash	groundnut	
8:50	MM	BB	Mother giving raw groundnut to the baby	groundnut	
8:55	MM	BB	Mother giving water to the baby	water	
9:00	MM	BB	Mother breast feeding the baby	Breast milk	
9:05	MM	BB	Mother breast feeding the baby	Breast milk	
9:15	BB		Baby playing with his mother		
9:20	MM	BB	Baby sitting on his mother lap		
9:25	BB		Baby eating groundnut		
9:30	MM	BB	Mother breast feeding the baby	Breast milk	
9:35	MM	BB	Mother breast feeding the baby	Breast milk	

TIME	ACTOR INITIALS	RECIPIENT INITIALS	WHAT HAPPENED	FOOD	Reactivity
9:40	MM	BB	Still breast feeding	Breast milk	
9:45	BB		Baby is sleeping on his mother lap		
9:50	BB		Still sleeping		
9:55	BB		sleeping		
10:00	MM	BB	Mother sleeping with the baby		
10:05	FF		Father coming back home with a hoe and a bag		
10:10	FF		Father going out with a bike with the hoe on his back		
10:15	BB		Baby still sleeping		
10:20	MM	BB	Mother breast feeding the baby	Breast milk	
10:25	MM	BB	Breast feeding continue	Breast milk	
10:30	BB		Baby sleeping on his mother back		
10:35	BB		sleeping		
10:40	BB		sleeping		
10:45	BB		Sleeping		
10:50	HDI		The husband of the daughter in-law came in to drop a hoe and a bag		
10:55	BB		Baby still sleeping		
11:00	MM	BB	Mother breast feeding the baby	Breast milk	
11:05	MM	BB	Mother breast feeding the baby	Breast milk	
11:10	YB, SB,SG,SGS		The children came back with hoe and a bag.<br>They are eating tuwo dawa (sorghum) with dried baobab	Tuwon dawa with baobab soup	

TIME	ACTOR INITIALS	RECIPIENT INITIALS	WHAT HAPPENED	FOOD	Reactivity
			soup. Young boy eating alone, the small girl with her sister eating together		
11:15	SG	BB	The small girl is giving the baby the food	Tuwon dawa with baobab soup	
11:20	YB, FF		The oldest boy and the father went out		
11:25	BB		The boy is eating tuwon dawa with baobab soup	Tuwon dawa with baobab soup	
11:30	SG, SGS		The small girl went out to fetch water and her sister follow her		
11:35	MM	BB	The mother brought pap in a bowl, open the MNP and pours it into the pap, stir and give the baby. He didn't like the MNP, she gave he bowl the baby drank small and leave the remaining. The mother said let me keep it may be he just eat tuwo, he is not hungry. She now breast feed him.	Cold pap. The pap is semi solid but the baby couldn't finished it	She was looking at me while administerin g the MNP
11:40	MM	BB	Breast feeding continue	Breast milk	
11:45	BB, SGS		Baby playing with his sister		
11:50	MM		Mother washing clothes		
11:55	DI	BB	Daughter in-law backing the baby		
12:00<br>12:05<br>12:10	DI, SGS	BB	The baby starts crying, she bring him and down and brought the bowl of pap in front of him and starts drinking it. The youngest daughter took the remaining and drank. The baby starts crying and the daughter in-law took him and back him then the	Pap and MNP	

TIME	ACTOR INITIALS	RECIPIENT INITIALS	WHAT HAPPENED	FOOD	Reactivity
			mother came and take him from her and breast feed him		
12:15	BB	MM	Baby playing with his mother		
12:20	MM	BB	Mother breast feeding the baby	Breast milk	
12:25	MM	BB	Mother put the baby on her feet with his two legs open he started stooling. The mother washes his body and put dry sand on the stool, then uses iron shovel to pack it outside the fence		
12:30	MM	BB	Mother backing the baby		
12:35	MM	BB	Mother breast feeding the baby and she is standing		
12:40	DI		Daughter in-law bring millet and starts pounding, washes the grain and removing the chaff and then put a little water into the grain and keep		
12:50	BB		The baby is playing		
12:55	BB,SBS		Baby playing with his sister		
1:00	BB		Baby playing		
1:05			Baby playing		
I:10			Baby playing		
1:15	MM	BB	Mother breast feeding the baby	Breast milk	
1:20	MM	BB	Mother breast feeding the baby	Breast milk	
1:25	SGS, SG		The small girl and her sister are drinking pap together	Semi solid pap	
1:30	BB		Baby is playing with his sister		
1:35	BB		Still playing		

TIME	ACTOR INITIALS	RECIPIENT INITIALS	WHAT HAPPENED	FOOD	Reactivity
1:40	BB,MM	BB	Baby starts crying and the mother breast feed her	Breast milk	
1:45	MM	BB	Breast feeding continue		
1:50	MM, SG		Mother and daughter are pounding maize, washed the maize with water to remove the chaff and put a little water into it (grain). The washed water of the maize and millet were add together for cow, sheep to drink in a bowl.		
2:00	BB,DI,MM		Baby was crying and the daughter in-law gave him water and he continue crying and the mother breast feed him and back him		
2:15	SGS		The youngest daughter is drinking pap and the baby starts crying, the mother kept him and the sister with the pap bowl, he then starts drinking the pap		
2:20			The baby and the sister are playing		
2:25	DI		Daughter in-[law is pounding the grains (millet) until its turns to flour, sieve it, add water into the flour and pound again until it turn to paste then she put the paste in a calabash and keep		
2:35	MM	BB	The mother is breast feeding the baby		
2:40	MM	BB	The mother back the baby he starts crying, she then bring him down and breast feed him		
2:45	BB		Baby on his mother back		
2:50	BB		Baby sleeping on his mother back		
	MM		Mother and daughter are		

TIME	ACTOR INITIALS	RECIPIENT INITIALS	WHAT HAPPENED	FOOD	Reactivity
2:55			separating the baobab leaves, removing the bigger leaves from the smaller ones		
3:00	BB		Baby sitting on the flour		
3:05	BB		Sitting on the flour		
3:10	BB		Baby playing with the sister		
3:15	SG	BB	Sister giving the baby water	Water	
3:20	BB		Baby playing with the sister		
3:25	DI	BB	Sister giving the baby water	Water	
3:30	BB		Baby eating the millet paste	Millet paste	
3:35	DI	BB	Daughter in-law give the baby water	Water	
3:40	DI	BB	Daughter in-law add the paste to the baby		
3:45	DI	BB	Daughter in-law washes his hand and carry him		
3:50	SG	BB	The sister drank water and also gave the baby	Water	
3:55	MM	BB	The baby sees the mother and starts crying, she took him and breast feed him		
4:00	MM	BB	Mother breast feeding the baby	Breast milk	
4:05	MM	BB	Mother playing with the baby		
4:10	SB		The small boy came in, took the bowl and fetch pap and drank	Cold semi solid pap	
4:15	BB		Baby is playing on the ground		
4:20	FF	BB	Father came in take the baby and said to me ‘I hope you are managing with our house, you know we are villagers’ enter the room and spent some times and		The father came into the house because I was there

TIME	ACTOR INITIALS	RECIPIENT INITIALS	WHAT HAPPENED	FOOD	Reactivity
			went out		
4:25	BB		The baby is playing on the ground		
4:30	MM	BB	The baby starts stooling and the mother took him and washes his body then back him. She then put sand on the stool and uses shovel to pack it outside the fence		
4:35	MM	BB	Mother breast feeding the baby	Breast milk	
4:40	MM	BB	Breast feeding continue	Breast milk	
4:45	MM	BB	Mother gives him millet paste on his hand and he eats	Millet paste	
4:50	MM	BB	Mother gives the baby water from the kettle		
4:55	MM	BB	Mother breast feed the baby, the father came in and ask of water in a kettle, she now went on her knees and handed the kettle to him	Breast milk	
5:00	BB		Baby is playing with his sister		
5:05	MM		Mother setting the fire		
5:10	BB		Baby is crying		
5:15	MM,SG		Mother sent her daughter to grind the maize they pounded earlier		
5:20	MM		The mother wash the pot and add water, set the pot on fire and closed the pot		
5:25	MM	BB	Mother breast feeding the baby	Breast milk	
5:30	BB		Baby playing		
5:35	DI		Daughter in-law is washing the dishes		
5:40	MM	BB	Baby is crying following the	Breast milk	

TIME	ACTOR INITIALS	RECIPIENT INITIALS	WHAT HAPPENED	FOOD	Reactivity
			mother, the mother now took him and breast feed him while standing		
5:45	BB		Baby playing		
5:50	MM		Mother takes small from the boiling pot and add water. She use the water to bath the baby	Breast milk	
5:55	MM		Mother sieving the grinding maize		
6:00	MM		Mother took little from the maize flour, add cold water into the remaining stir it with her hand and pour into the boiling pot and stir, she close the pot		
6:05	MM		The baby starts stooling on the ground, the mother took him wash his body and give him water		
6:10	SG, SGS		The two sisters are drinking pap	Semi liquid cold pap made from millet	
6:15	BB		Baby drinking pap	Cold millet pap	
6:20	MM		Mother setting fire and put a small pot on the fire then add water then close the pot		
6:25	MM	BB	Baby starts following the mother and crying then the mother took him, give him water and breast feed him	water	
6:30	DI		Daughter in-law sleeping on the mat		
	MM	BB	Mother breast feeding the baby	Breast milk	

Primary Questions (Complete <u>after</u> you complete data collection):

From what you have seen observing THIS HOUSEHOLD, please answer the following questions in detail:

Question	Write your <u>detailed</u> response here
1. In this household, what would be the best way(s) to give nutritional supplements such as an MNP to young children to ensure compliance?<br><br>Why?	Is through the mother because the mother takes care of the children.<br>Secondly, this household the daughter in-law help in taking care of the children especially the baby, she help her mother in-law in all the activities of the house including the meal preparation.
2. In this household, who is favored during meal preparation or feeding?<br><br>How is this favoritism expressed?	The baby is favoured because he is protected by his mother, sisters, father and the mother in-law, they give him whatever he wants
3. How could we promote the MNP supplement for this family that would attract their attention?<br><br>Who would they listen to?	Through the head of the house that is the father because he is in total control of the house. He provide for the family and they listen to him.<br>The mother serve the husband, the moment he comes back she stop whatever she is doing and attend to him. He asked of water in the kettle she knelt down before giving him. She wished her husband well when he is going out.
4. In this household, what would be the best ways to promote the MNP but limit how much it would be shared?<br><br>Why?	Is through proper sensitization because from what I gathered they don't have proper/enough information, rules and regulation about the product because when the daughter took the remaining pap of MNP, the mother didn't stop her or show concern that the daughter is taking the MNP pap
5. What other issues and concerns did you see with this family that are important for us to consider with regard to introduction of an MNP?<br><br>Why?	1. Hygiene, their room, surrounding, compound, dishes are very dirty, animals all over, animal faeces all over even in their kitchen. Chicken laying eggs under the bed in short the house is very dirty.<br>2. Sharing of MNP<br>3. Carelessness, because they allowed the goats to be eating their grains that was kept within their reach
6. Based on your experience observing this child today, do you believe reactivity negatively impacted your ability to see the food-related behaviors of the	Reactivity didn't impact negatively to my ability to see the food related behaviours of the child, because they relaxed and even tend to forget about my presence in the house. The only place was during MNP administration, they told me that they were told that a monitor will come to observe them, so I think this is the reason for the reactivity

child?<br><br>Please explain your answer in detail.	
